# Unaltered fungal community after fire prevention treatments over widespread Mediterranean rockroses (*Halimium lasianthum)*

**DOI:** 10.1038/s41598-023-27945-1

**Published:** 2023-01-12

**Authors:** Pablo Martín-Pinto, Cristina Fernández, María Santos, Teresa Fontúrbel, Juan Andrés Oria-de-Rueda, Aitor Vázquez-Veloso, Tim Stadler, Olaya Mediavilla, Ignacio Sanz-Benito

**Affiliations:** 1 Sustainable Forest Management Research Institute UVa-INIA, Avenida Madrid, S/N, 34004 Palencia, Spain; 2 Centro de Investigación Forestal-Lourizán, Xunta de Galicia, P.O. Box. 127, 36080 Pontevedra, Spain; 3 University for Sustainable Development Eberswalde, Schickler Street 5, 16225 Eberswalde, Germany; 4 Department of Vegetal Production and Natural Resources, University of Valladolid, Avenida Madrid, S/N, 34004 Palencia, Spain

**Keywords:** Forest ecology, Forestry

## Abstract

Mediterranean ecosystems are frequently invaded by pyrophytic scrubs such as *Halimium lasianthum* that colonize areas traditionally used by livestock. A diverse fungal community is associated with this kind of vegetation, playing an important ecological role in these ecosystems. However, uncontrolled expansion of these shrubs considerably increases the risk of wildfires in these stands and, hence, fire-prevention treatments are needed. To investigate the long-term effects of two different forest-fire-prevention treatments on the soil fungal community, we analyzed these communities 9 years after prescribed burning or mechanical shredding were carried out in scrubland dominated by *H. lasianthum*. Neither of the fire-prevention treatments had a negative long-term effect on the abundance or richness of ectomycorrhizal fungi. However, saprotrophs and lichenized fungi experienced negative effects. Soil fertility significantly affected the distribution of fungi according to their functional groups, and pH was the most influential variable in terms of the distribution of edible species. Our findings indicate that forest management practices to prevent forest fires does not negatively affect the fungal community in the long-term, but for lichens and decomposers. Moreover, prescribed burning is suggested as a more economical way of reducing the risk of wildfires without affecting the ecology of the fungal community.

## Introduction

Forest fire is one of the major factor in forest resource degradation and affect fungal communities, primarily through fuel accumulation on the forest floor^1^. The need to reduce fire risk in the forests system while minimizing adverse impacts to the resource is an important challenge for forests managers, especially in fire-prone areas. In this regard, to prevent the negative effects of fires, fuel reduction treatments (mechanical and prescribed burning) are commonly used in the forests systems^2^ as they are also used as part of habitat management or ecological restoration^3,4^. Of these technics, the mechanical mastication is attractive as a fire surrogate to reduce fuels because it is cost effective and efficient^5^, while the prescribed fires are usually applied at low intensity and severity and generally does not have a significant impact on the forest resources^4^. However, the potential adverse effects of both the mechanical and prescribed fire treatments are a concern to forests managers because information about the ecological consequences are still lacking^5^.

Interest in the Cistaceae family is increasing due to its relationship with the increasing frequency of wildfires and its encroachment of former agricultural land. Aromatic compounds produced by the Cistaceae promote fire^6,7^ and the germination of Cistaceae seed is induced by heating, which happens in a wildfire, stimulating the rapid colonization of Cistaceae in burned areas^8,9^. However, this type of scrubland has also been generating interest because of its relationship with fungal succession and common mycorrhizal networks^10,11^. One of the less-studied genera among the Cistaceae family is *Halimium* spp., which is widely distributed throughout the Mediterranean region in degraded forest, open sites and even dry dunes^12^ from Portugal to Lebanon, and is even found as far north as Belgium^13,14^. *Halimium* is even found, like its *Cistus* relative, play a key ecological role as a dual-mycorrhizal plant helping in the fungal succession, particularly after fire disturbances^14^. The life cycle of this species is approximately 20 years^15^, which means that this genus shows rapid regrowth, enabling long- or mid-term studies of *Halimium* stands to be performed after a disturbance.

Traditionally, the expansion of this kind of scrubland was controlled by cattle grazing^16^, which was a natural, efficient, and productive way of preventing forest fires by reducing the amount of forest fuel available. However, because fewer cattle now graze in these areas, more fuel is accumulating in these ecosystems. The lack of management of *Halimium* stands promotes an increased risk of wildfire, specifically crown fire which can affect neighboring forested areas due to the vertical continuity between shrubs and trees^17^ which would take decades to the forests stands to recover. All these factors can influence the forest resources by reducing the vegetation cover, changing plant composition and damaging fungal communities^10,18,19^, the latter being the focus of this study.

Fuel-reduction practices, such as prescribed burning or mechanical shredding^20^, need to be implemented to modify the quantity and continuity of fuels, and reduce the risk of high-intensity and severe wildfires^16^. On the one hand, prescribed burning causes less damage than wildfires^21^, and is considered a low-cost option^22^. In this case, methods for controlling the burn must be well established so as to minimize the damage to the forest ecosystem^23^. On the other hand, mechanical shredding is a safer technique than prescribed burning; however, it is more expensive^24^ and it is difficult to undertake on steep slopes and stony terrains^25^. Previous studies undertaken also with *Halimium lasianthum* in the north-west of the Iberian Peninsula, in the Galician region, with different objectives have compared the effect of prescribed burning and mechanical shredding treatments. However, those studies observed no differences in plant mortality between treatments, or in vegetation cover and recovery, or even in species richness, diversity, and evenness^24,26,27^. Although prescribed burning has been reported to remove all the litter, which creates appropriate sites for rockrose germination^28^ as it removes the plant material to control advance of fire close to the ground, colonization by rockroses did not affect the fungal diversity or community composition^29,30^. Moreover, it is suggested^31^ that prescribed fire fosters a diversity of mycorrhizal and saprotrophic fungi owing to the increase in pyrophytic species.

Given the importance of *Halimium* spp. as a host for ectomycorrhizal and arbuscular mycorrhizal fungi^14,32^, the goal of our study was to analyze soil fungal communities in *Halimium* scrubland nine years after prescribed burning or mechanical shredding were carried out (i.e., midway through the lifespan of these *Halimium* plants) to determine the long-term effects of fire-prevention treatments. First, we hypothesized that a high diversity of fungi would be found associated with the *Halimium* scrubland^14,15^. In addition, contrary to other long-term studies^33^, we secondly hypothesized that neither fire-prevention treatment (Prescribed burning and mechanical shredding) would have a negative effect on the total fungal richness^29^, which would support the implementation of the underused practice of prescribed burning in Southern Europe^35^. Furthermore, we hypothesized that none of the guilds would be affected by prescribed fire or mechanical" treatments, particularly ectomycorrhizal (ECM) fungi given that previous studies have reported that shrub recovery is successful in the short-term in this area^16,26^. Finally, in this sense, our fourth hypothesis was that the total and guilds fungal composition would be more affected by edaphic variables than by wildfire prevention treatments. Thus, we assessed the hypotheses through the following specific objectives: (i) to describe the soil fungal richness and diversity of *Halimium* scrublands in the study area; (ii) to evaluate the impacts of fire prevention treatments on soil fungal richness and on guild status and; (iii) to determine whether the soil fungal community composition was governed by wildfire prevention treatments and by the environmental factors of the study area.

## Results

### Taxonomic composition of fungal communities

**Figure 1:**
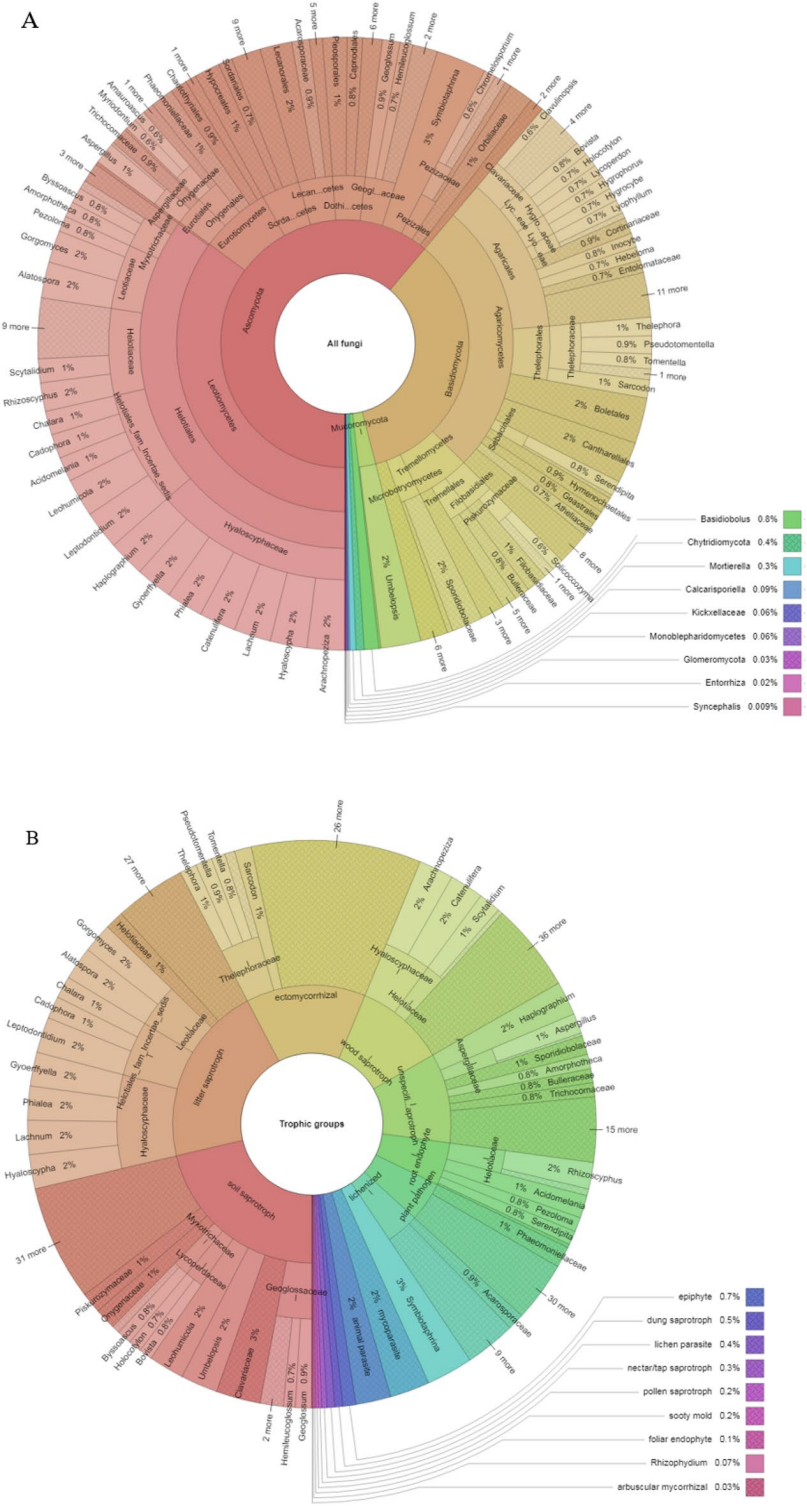
Krona charts showing fungal taxonomic classification in all treatments (prescribed, mechanical, and controls) at the phylum level (**A**) (name of phylum; number of OTUs; percentage); and (**B**) classification of fungal guilds based on the proportion of number of OTUs, including the taxa belonging to those guilds, based on Põlme et al.^45^.

A total of 2225 fungal OTUs were classified into 13 phyla and 329 genera (Fig. 1). Although most of the OTUs were identified down to genus level, it was not possible to identify all OTUs down to species level. The soil fungal community was dominated by Ascomycota, which were mainly represented by Leotiomycetes (34% of the total OTUs identified) and Basidiomycota, which were mainly represented by Agaricomycetes (21% of the total OTUs identified) (Fig. 1A). The Krona charts showing taxonomic classification of the ectomycorrhizal trophic groups found in all the treatments is given as supplementary figures (Sup Fig. 1).

In this study, 19 guilds were found, with saprotrophic guilds dominating the overall fungal community, including soil saprotrophs (13%) and litter saprotrophs (17%). There was also quite a large number of ECM fungal taxa (30 taxa: 3.7%) (Fig. 1B), including frequent taxa such as *Pseudotomentella*, *Thelephora*, and *Sarcodon* and other appreciated genera such as *Boletus*, *Terfezia*, *Amanita*, *Russula* or *Rhyzpogon* (Fig S1).

### Effect of fire-prevention treatments on fungal richness

**Figure 2:**
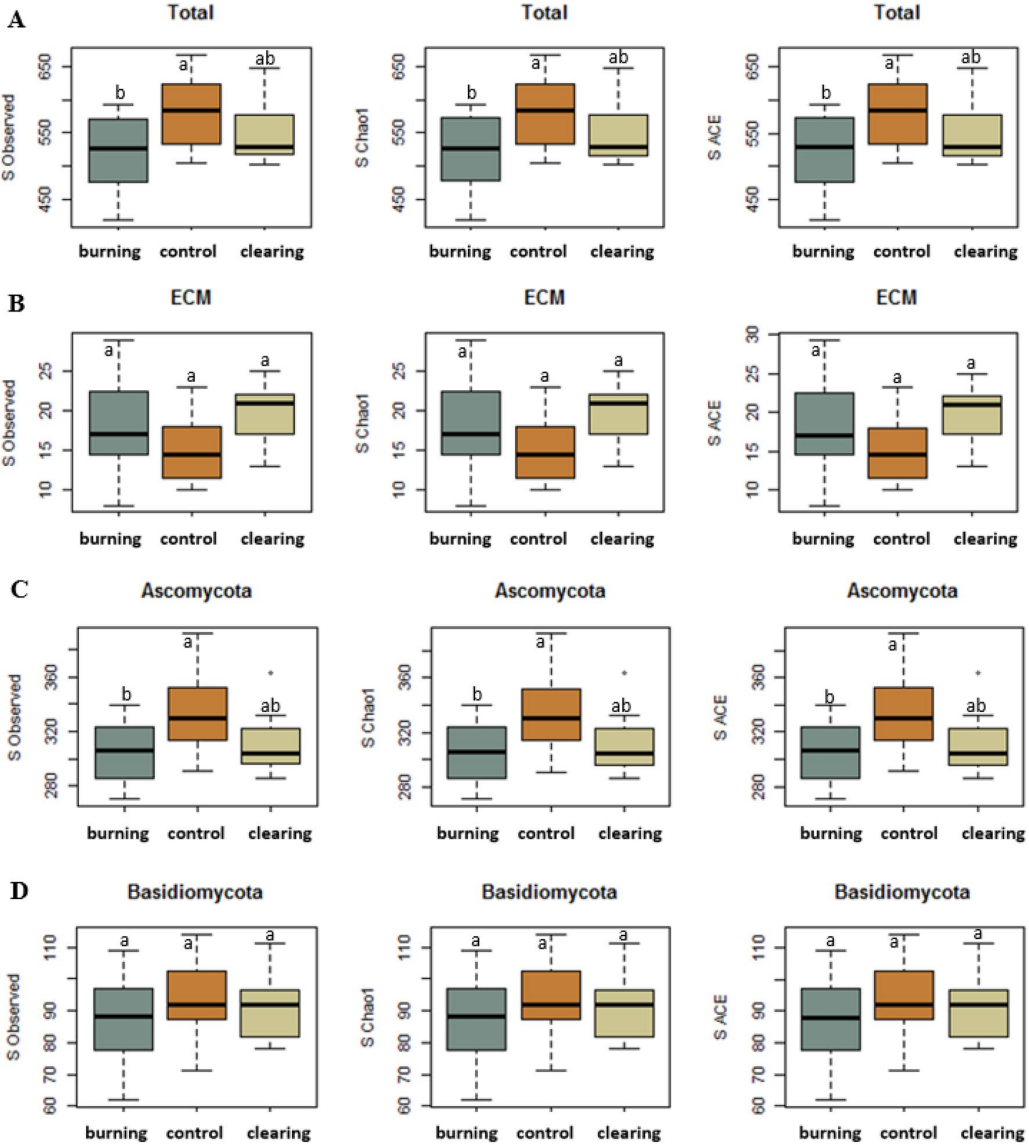
Proportional species richness and richness estimation of total (**A**), trophic guilds (**B**) and fungal phyla (**C,D**) by extrapolating the singleton and doubleton (Chao1) values or rare species (ACE, abundance-based coverage estimator; abundance < 10 species) in soil samples collected 9 years after scrublands were subjected to prescribed burning, mechanical clearing or no treatment (Control). Different lowercase letters above bars indicate a significant difference between treatments (*p* < 0.05).

Species richness estimates calculated using Chao1 and ACE were higher than the observed richness values, indicating a low underestimation bias (Fig. 2). Accordingly, the observed species richness values are used from here onward to represent the soil fungal community.

There were significant differences in terms of observed total fungal richness between treatments (*p* = 0.034) (Fig. 2A). The richness between the control and the burned plots differed significantly (*p* = 0.027), but not between mechanical shredding and control (*p* = 0.231). Moreover, Ascomycota phyla significantly differed in richness between treatments (*p* = 0.024), which also reflected differences in richness between the control and the burned plots (*p* = 0.026), but not between control and mechanical shredding (*p* = 0.08) and much less between burned and mechanically crushed stands (*p* = 0.851) (Fig. 2C). No differences between treatment plots were found for the other main phyla (Fig. 2D).

An unexpected lack of differences among treatments was found among most of the trophic guilds, including the ECM fungi (Fig. 2B). Lichenized and saprotrophic fungi showed significant differences between treatments (*p* < 0.01) (Table 1). However, Lichenized fungi was richer in the control plots than in the cleared (mechanical shredding) and burnt plots (*p* < 0.01, in both cases), but not between treated plots (*p* = 0.85). There was also significant difference between treatments in terms of saprotrophs fungi, with higher richness in the control plots than in the burnt plots (*p* < 0.001) or in the cleared plots (*p* = 0.03), but not between treated plots (*p* = 0.76).

**Table 1:** *F* and *p* values for LME comparisons of guilds among treatments.

Fungal guild	Richness	
	*F*	*p-value*
Animal pathogen	1.536	0.23
Arbuscular mycorrhiza	2.218	0.125
Ectomycorrhiza	2.766	0.077
Endophyte	1.087	0.349
Ericoid mycorrhiza	1.575	0.222
Lichenized	9.345	***p* < 0.01**
Orchid mycorrhiza	0.178	0.837
Plant pathogen	3.144	0.056
Saprotroph	6.112	***p* < 0.01**
Unknown	2.412	0.105

*Significant *p* values are shown in bold.

### Ecological factors driving fungal community composition

**Figure 3:**
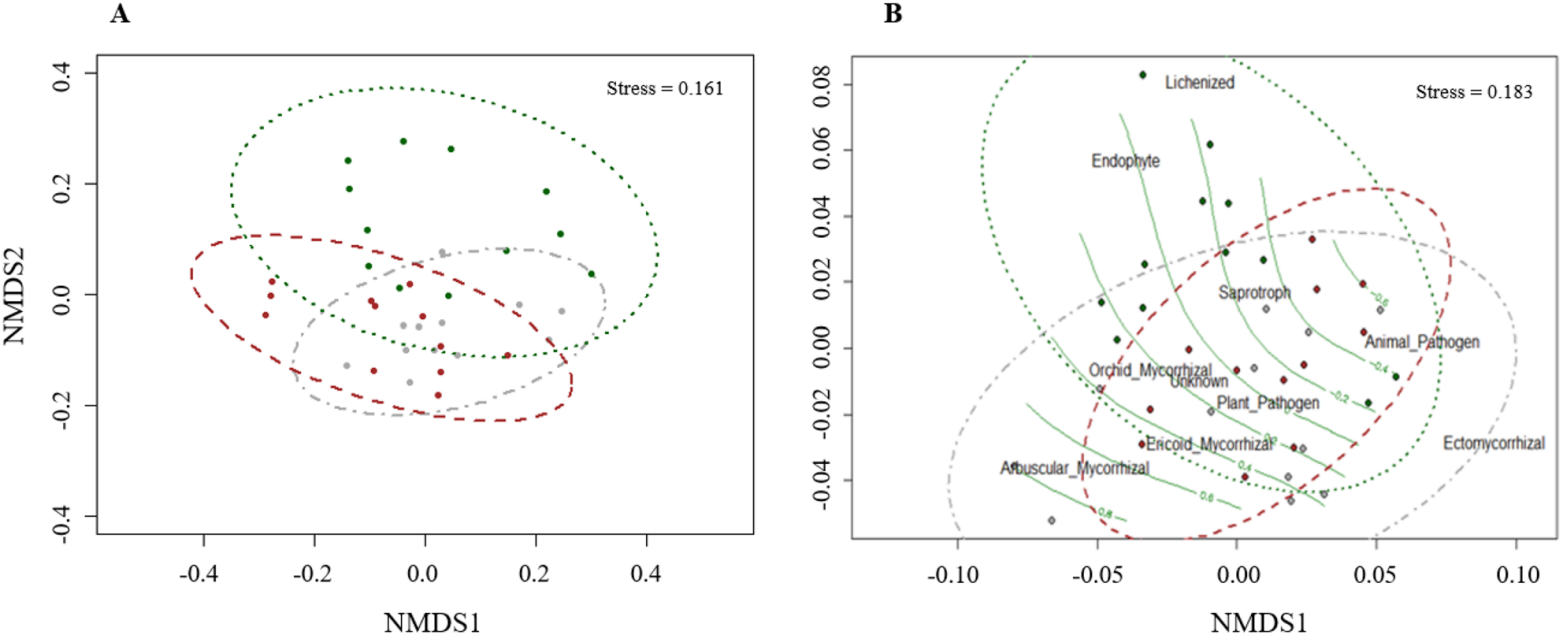
Non-metric multidimensional scaling (NMDS) of total fungal taxa (**A**) and trophic guilds (**B**) in soil samples collected 9 years after scrublands were subjected to fuel-reduction treatments. Treatments: control (dark green), burned (dark gray), cleared (brown). Ellipses represent the distribution of the fungal community related to the treatment. Isolines of the gradient of the C content (**B**) were also plotted on the NMDS ordinations using the *ordisurf* Model Gaussian function.

The analysis of the total community showed a stress value of 0.161, indicating weak ties with no significant differences between treatments (*p* > 0.05). The distribution of the community did not show differences between treatments (Fig. 3A).

The analysis of guilds distribution showed weak ties with a stress for the model of 0.183 again. No significant differences were found between the treatments (*p* > 0.05), with pH being the unique influential environmental factor in our analysis (*p* = 0.026; *r*^*2*^ = 0.214) (Table 2). The distribution of trophic guilds (Fig. 3B) in the control was insignificantly different than the burning or mechanical shredding treatment, which tended to be more similar. The fungal communities in the control plots included more lichenized and endophytic fungal taxa, whereas ECM and arbuscular mycorrhizal fungal taxa were more associated with burnt plots. The soil fungal community in mechanically shredded plots, which was at the core of the shared community, appeared to occupy an intermediate position between burned and control plot communities.

**Table 2:** Summary of *p*-values of the environmental variables influence over the three treatments fungal community distribution in the NMDs analyses.

Variable	Taxa	Guilds
pH	0.001***	0.026*
P	0.157	0.156
N	0.001***	0.320
C	0.001***	0.236
Dry matter	0.003**	0.219
Cover	0.583	0.682

## Discussion

### Effect of fire-prevention treatments on fungal richness

Reports on soil fungal species from *Halimium* scrubland are limited. As to our knowledge, this study is the first to explain fungal communities in the *Halimuum* scrubland of the Edreiras mountains in Ourense province, North west Spain. As expected, *Halimuum* scrubland was associated with large number of fungal spies as we found a total of 2225 fungal OTUs, belonging to 329 genera and 19 different guilds, with saprotrophic composition dominating the overall fungal community. This was an indication of the importance of *Halimuum* scrubland in terms of diversity of soil fungal species in the study area. The present study also revealed occurrence of valuable ECM genera such as *Boletus*, *Terfezia*, *Amanita*, *Russula* and *Rhizopogon* among others. We also, in this study found that the total soil fungal richness seemed to be affected by prescribed burning treatment as compared to the control due to its effect on Ascomycetes, more specifically saprotrophic fungal richness, agreeing with other studies^36^. It is assumed that after a fire, the new ecological conditions lead to a rapid dominance of pyrophilous (Fire loving) ascomycetous species that later stabilize to form a Basidiomycota–Ascomycota community^37^, which is possibly due to the availability of a large amount of decomposable material following the fire^38,39^. The two treatments didn’t show a significant difference for the comparison of total fungal richness as expected in the second hypothesis, with the overall fungal community unaltered by the fire-prevention treatments in terms of composition at the large scale, and only insignificant altered in terms of richness.

In line with our hypothesis, the treatments barely affected the richness of trophic guilds. However, significant differences in the richness of two types of guilds (Lichenized and saprotrophic fungi) were observed between the control plots and the both types of treated plots. Like Ascomycota, saprotrophic fungi were similarly affected by both treatments. Different studies have reported contrasting effects of fire on saprotrophs: beneficial^29^, determinantal^36^, or neutral^40^. This suggests that the reaction of this guild to fire is context-dependent. A previous study performed at our experimental site recorded a decrease in the depth of the soil organic layer after prescribed burning^24^, which possibly inhibited the subsequent development of the saprotrophic community in the short-term given that it is often found to dominate the uppermost soil layers in forest ecosystems^41^. However, the ability of certain fungi to form heat-resistant structures contradicts this previous assumption given that the survival of thick-walled sclerotia or spore banks might give the surviving communities a competitive advantage^36^. It could be suggested that the low severity of our prescribed burning had yet bigger effect than expected over the soil organic material in comparison to the control. However, not enough to be differentiated from the mechanical shredding effect, which still leaves plant remains to be decomposed, bringing the possibility to an effect more related to other environmental factors than just the organic decomposable material. In the context of a fire, the woody biomass that normally hosts saprotrophic species is reduced in postfire forests^42^, which ultimately reduces the habitat that these organisms need given that it is a scrub-dominated vegetation. Furthermore oak-dominated areas subjected to prescribed burning produce litter with a higher C:N ratio than scrublands, leading to a slower decomposition rate^43^ and an altered saprotrophic community. Short-term experiments have shown that fire negatively alters the richness and abundance of saprotrophic fungi^44^, suggesting that the saprotrophic part of the community structure might require more than a decade to recover to its pre-fire state^45^. The fungal community associated with the mechanically cleared plots differed from those reported in previous studies^34,46^. Changes in the richness of saprotrophic fungi may be due to changes in the micro-environmental characteristics of the site, which alter the temperature, humidity, and light exposure of the area, negatively affecting the inhabiting community^47,48^. These alterations can also negatively affect cyanolichens and crustose sorediate species of lichens due to the reduced canopy cover, which is crucial for their survival^49^. However, this argument needs empirical research to justify so that we recommend further studies related to these issues. Lichenized taxa lost in this way might take years or decades to fully reestablish^50^. However, the resprouting behavior of plants from buried roots gives understory plants an advantage in terms of reassembling their fungal community in postfire forest ecosystems^36,51^. Previous studies performed at the experimental site^24,27^, as well as other studies^36^ have reported a high survival rate of resprouting plants following a fire. Therefore, we suspect that surviving roots buried in the soil might protect mycorrhizas and fungal endophytes from fires suggesting that vegetation communities consisting of plants able to resprout after fire could be considered more suitable for prescribed burning practices than other types of vegetation. Based on the assumption that there is a close ecological relationship between the vegetational and soil fungal communities, the relatively rapid recovery of the plant community after a fire treatment, as observed in earlier studies performed at the experimental site^24,26,27^, might have a beneficial effect on the recovery of the soil fungal community. In general, most of the phyla and guild richness levels were not affected by either of the fire-prevention treatments, potentially giving forest managers the option of using the fire-prevention treatment that is most appropriate for the characteristics and conditions of the area^20^. Although neither fire-prevention treatment appeared to have any severe long-term effects on the soil fungal communities assessed in this study, the deterioration of the soil fungal community in the short-term must be considered^52^. In particular, C- and N-stabilizing ascomycetes fungi might suffer from repetitive burnings over short time intervals, leading to a reduced capacity for C sequestration given that these fungi become more abundant in the long-term after fire events^53^.

### Relationship between ecological factors and fungal community composition

Ecological factors have been observed to have non-significant effects on the taxa community^29^. However, in the case of guilds, the effects of treatments showed a more noticeable influence over the guilds distribution than the taxonomic one. The only environmental variable that showed significant effect over the guild distribution was the pH (Table 2), which is in agreement with others studies^36,39,54,55^, which could influence indirectly through plant diversity and mold prevalence, as through the solubility of minerals as P^56^. Although, soil pH levels recover to the pre-fire state through time when applying prescribed burning, however it is possible that our burned stands still experience a higher pH due to the ignition of organic acids^57^ during post-fire colonization, influencing the distribution. The long-term effects of prescribed burning on lichens tend to last for 10 to 15 years after treatment^58^ as well as alterations to micro-environmental conditions that affect them negatively^49^, such as canopy cover lost, relative to those growing in control stands. In the case of the endophytic population, fire-prevention treatments could lead to a change in guild status (i.e., endophyte to saprobic or pathogen)^59^, supporting the ‘body snatcher fungi’ hypothesis, which suggest that endophytes change their status after a disturbance adopting a new kind of functional role^60^.

In the long term, ECM fungi tend to recover after a fire of medium or low severity^37^ as the remaining C and N begin to becomes more recalcitrant, which negatively affects the saprotrophic fungi, thus, enabling ECM fungi to become more dominant^39^. Species colonization, such in the case of *Rhizopogon* spp., can be fostered by heat treatments^61^. This could be related to heat-resistant spores^62^, allowing mycorrhizal fungi to assist rapidly in the reforestation^63^, or the creation of new niches occupied by ECM fungi^64^, thanks to their spore production capacity^65^. Arbuscular mycorrhizal fungi also appeared to be associated with this forest type, which may be related to the existence of remaining prairie areas in burned stands^66^ or to the high resilience of arbuscular mycorrhizal fungi when facing fire disturbances in the long-term^67^. Furthermore, some studies have shown an increment in the relative abundance of mycorrhizal fungi based on analyses of spores or sequences^68^. As the differences in terms of composition are quite discrete and the overall fungal composition did not differ significantly between treatments, either fire-prevention treatment could be used. Other ecological or socioeconomic factors could also be a key factor when selecting a fire-prevention treatment^69^.

## Conclusions

This study was carried out in *Halimium* stands in an area where the information in relation to fire prevention treatments and soil fungi is limited. Thus, this work could provide valuable scientific insight to promote the use of fire prevention treatments for the conservation fungal resources in the study area. In this study, in accordance with our expectation, many fungi are found in association with *Halimium* stands, indicating the importance of these stands in terms of fungal diversity. Also, no differences were found in terms of richness between treatments, and the taxa detected under the different treatments were almost identical. However, our expectation that the fungal guilds would not be significantly affected by the fire-prevention treatments was not entirely confirmed because the treatments did have a negative effect on the richness of saprobic ascomycete fungi and lichens; however, the richness of ECM fungi was not negatively affected by either of the treatments and was even promoted compared with the richness of the control stands (although not significantly). Furthermore, the fire-prevention treatments would not have a negative impact on the fungal community was confirmed as expected, with the overall fungal community unaltered by the fire-prevention treatments in terms of composition at the large scale. The promotion of a discrete mycorrhizal characterization was observed in burned plots in the community composition analysis, suggesting that the ability of ECM fungi to support forest succession was not affected by this fire-prevention treatment. This finding supports the idea of using non-spread prescribed burning treatments in certain regions of the Mediterranean basin where these *H. lasianthum*-dominated stands can be found. The lower cost of prescribed burning compared with mechanical clearing is another advantage, reducing the already high costs required for wildfire prevention. Moreover, further research should be carried out to investigate how the fungal response correlates with other ecological factors involved in this ecosystem, and possibly link it to the sustainable use and management of this ecosystem.

## Methods

### Study area and experimental design

The study site is located in the Edreiras mountains (42° 8′ 02″ N–7° 26′ 17″ W, 1330 m a.s.l.), in Ourense province (NW Spain). It experiences Mediterranean climate conditions with an average rainfall of 1100 mm year^−1^ and a mean annual temperature of 10 °C. The soils are characterized as Alumi-umbric Regosols (FAO, 1998) developed on schists, and the sites, on average, have a 10% slope. The experimental area is mainly covered by heather, the main species being *Erica australis* L., together with *Pterospartum tridentatum* (L.) Wilk. and *Halimium lasianthum* (Lam.) Spach. Cattle and roe deer are very frequent in this area and are two important agents of ecosystem changes. In the past, prescribed burning was commonly motivated by pastoral considerations. For further information about this study area, see previous studies in this study area that investigated shrub recovery^16^ and resprouting^24^, seedling emergence and microbial responses^70^ to fuel reduction treatments.

Plots were established in the same local area to reduce the influence that differences in altitude^71^, aspect^72^, slope^73^, and climatic conditions^74,75^ may have on different fungal communities. The experiment involved the analysis of two different fire-prevention treatments to obtain different plot conditions, i.e., plots that had either been burned by prescribed burning or cleared by mechanical shredding in 2010, as well as control plots in which vegetation had been growing naturally since 2003 when the last wildfire affected the study area. A total of four experimental blocks were established. Each block was composed of nine rectangular plots, with 50 × 16 m^2^ plots (800 m^2^) per treatment (i.e., burned, cleared, and control plots), to reduce the influence of variations in vegetation and stoniness. A total of 36 plots were surveyed.

### Treatments

The following parameters were used to choose the meteorological window according to Fernandez et al.^2^: a temperature of 20 °C, a relative humidity range of 40 to 60%, and a low wind speed of 2 m/s. For the high dead fuels, the required moisture content was between 10 and 15%. For the organic layer, it was > 80%. The amount of soil moisture was almost at field capacity. With a strip width of 10 m, plots were burned using a strip head fire. The fires were captured on videotape, and the flame length was calculated by comparing it to stakes that were placed at various points across the plot and were known to be that length.

A steel-track tractor was used to masticate the above-ground biomass into a patchy layer of small diameter woody debris that was about 5 cm thick and stayed on the soil surface. In order to achieve treatment homogeneity, the operator made deliberate passes through the vegetation. Visual inspections revealed that the root systems and beneath soil were unaltered. The shrub clearance procedure involved manually cutting shrub with a trimmer from the base of the plants and removing it from the parcels. In our case, there was a greater than 95% clearance of vegetation.

### Soil sampling and molecular work

After removing superficial stones and roots, 15 soil core samples were collected per plot between May and June 2019. The samples were mixed to form one pooled soil sample per plot to preserve the heterogeneity of the terrain, i.e., 36 pooled soil samples in total. The samples were placed in labeled plastic bags, transported to the laboratory by using an ice bag, and then processed within 24 h. Samples were air-dried and sieved through a 1-mm mesh sieve before analyzing them to remove rocky and oversized soil material. Each sample was subjected to genomic DNA analysis, which was performed using 0.25 g of soil which was stored at 4 °C for until subjected to DNA extraction. Soil physicochemical analysis was performed using two 20 g samples of each soil sample. Soil pH was determined by water-based suspension at 1:2.5. Also, to determine dry matter (%) following UNE-ISO 11465 rule and total phosphorus (P) using the Olsen methodology. For total carbon (C) and total nitrogen (N) contents (%) the Dumas methodology was used (Table 3).

**Table 3:** Mean physicochemical properties of soil samples collected 9 years after scrublands were subjected to prescribed burning or clearing (mechanical shredding) treatments.

Soil properties	Treatment		
	Burned	Cleared	Control
pH	4.49 ± 0.06 a	4.43 ± 0.07 b	4.46 ± 0.04 ab
P (mg/kg)	9.32 ± 2.19a	12.08 ± 3.53 b	9.98 ± 2.86 ab
N (%)	0.65 ± 0.05 ab	0.68 ± 0.04 a	0.62 ± 0.07 b
C (%)	10.47 ± 1.11 ab	11.30 ± 0.88 a	9.94 ± 1.52 b
Dry matter (%)	95.18 ± 0.68 a	94.27 ± 1.38b	95.97 ± 0.55 a

*Different lowercase letters indicate significantly different mean values (*p* < 0.05).

A PowerSoil™ DNA Isolation Kit (MoBio Laboratories Inc., Carlsbad, CA, USA) was used to extract DNA from 0.25 g of soil per sample. PCR reactions of each sample were carried out in triplicate to minimize PCR biases. PCR reactions were performed in 20 μL reaction volumes containing 11.22 μL of Modified Quantization (MQ) water, 1.60 μL of DNA template, 2.00 μL of 10 × buffer, 1.40 μL of MgCl_2_ (50 mM), 1.60 μL of dNTPs (10 mM), 0.50 μL of bovine serum albumin (2%), 0.80 μL of reverse and forward primers (10 μM), and 0.08 μL of Platinum Taq polymerase (Invitrogen, Carlsbad, CA, USA). The following PCR conditions were used: an initial denaturation step at 94 °C for 3 min; followed by 35 cycles of 94 °C for 45 s, 50 °C for 1 min, and 72 °C for 1.5 min; and finally, one cycle of 72 °C for 10 min. PCR was used to amplify the ITS2 region (ca. 250 bp) of the nuclear ribosomal DNA repeat using primers fITS7^76^ and ITS4^77^, as described by Geml^71^. Sample-specific Multiplex Identification DNA-tags were used to label the ITS4 primer. A negative control comprising MQ water instead of DNA was included on each set of PCR replicates, which underwent PCR under the same experimental conditions and was shown to be amplicon-free on a gel. Sequencing was performed using an Illumina MiSeq platform (BaseClear BV, Leiden, the Netherlands).

Above-ground vegetation was surveyed by establishing a 50-m long vegetation transect in each plot. Plant cover and height were measured using a line interception methodology, as described by Kent ^78^.

### Bioinformatic analysis

We used cutadapt (Martin, 2013) to trim off poor-quality ends in both directions (3′ and 5′) using a quality criteria value of q = 15. The next step was to join both sequences of each sample using USEARCH v.10.0.240^79^ and cutadapt, with a minimum sequence length of 200 bp. Primers (ITS4 reverse and fITS7 forward) were trimmed and sequences with expected errors of > 1 were removed. Then, sequences were combined into a single sample and read count numbers were recorded to generate an operational taxonomic unit (OTU) showing the number of times that an OTU was detected in each sample. We assigned sequences to taxonomic groups based on their similarity to the curated UNITE database (version v.8.0 released on November 18th, 2018), which contains identified and unidentified sequences assigned to species hypothesis (SH) groups defined based on dynamic sequence similarity thresholds^80^. Excluding OTUs with < 70% similarity or with < 200 bp pairwise alignment length to a fungal sequence, a total of 2225 OTUs were obtained, representing a total of 292,098 quality-filtered sequences. Taxonomic studies generally only consider OTUs with > 95% identity; however, given that we were analyzing fungal communities from an ecological point of view, all the OTUs detected were considered to be of interest in this study.

Assignment of ecological guilds was performed using the PlutoF web workbench (https://plutof.ut.ee)^81^. OTUs with > 90% similarity to a fungal SH group with known ecological function were assigned to functional groups according to Põlme^82^. Some additional categories were assigned to the ectomycorrhizal (ECM) fungi following criteria used by Geml^73^, Põlme et al.^45^ and data published by Agerer^83^, Tedersoo and Smith^84^, and the DEEMY database (http://deemy.de).

### Statistical analysis

The statistical significance of variable groups under different treatments was calculated using a Monte Carlo permutation test (499 permutations). Soil variables, fungal proportional richness, and relative abundance were compared across treatments using Linear Mixed Effects models (LME, *p* ≤ 0.05), which were developed using the package Nlme^85^, where site was defined as random and fire-prevention treatments were defined as fixed factors, and using Tukey’s HSD test. The R software environment was used for all these statistical analyses (version 3.5.3; R Development Core Team 2019). To avoid a downward-biased estimate of the species richness of the observed assemblage, two estimates, the Chao1 estimator and the abundance-based coverage estimator (ACE), were computed to extrapolate the singleton and doubleton values of rare species (abundance < 10 species), respectively, using the ‘estimateR’ function in the ‘vegan’ package^86–88^. The relationship between soil fungal composition and the edaphic variables was visualized using non-metric multidimensional scaling (NMDS) based on a Hellinger-transformed OTU and environmental scaled data matrix using the *metaMDS* function from the ‘vegan’ package. The effects of treatments were analyzed using a permutational multivariate ANOVA (PerMANOVA) based on 999 permutations using the *adonis* function in the ‘vegan’ package^89^. The correlation of NMDS axes scores with explanatory variables was assessed using the *envfit* function in R. The correlations of the environmental variables with the composition of the soil fungal community were determined by the Mantel Test using Bray–Curtis distance on the total species matrix and Euclidean distance on environmental parameters.

## Supplementary Information

Supplementary Figure 1.

## Data Availability

Provisional Submission number GenBank: SUB11719157. We state that we dispose all the data necessary to be required for proving the rigorous study here provided.
