# Supplementary material for: Unaltered fungal community after fire prevention treatments over widespread Mediterranean rockroses (Halimium lasianthum)
Source: Sci Rep. 2023 Jan 12;13:608. doi: 10.1038/s41598-023-27945-1 (PMC9837117; doi:10.1038/s41598-023-27945-1)
Supplement: Supplementary file 1 — Supplementary Figure 1. [file 41598_2023_27945_MOESM1_ESM.docx]

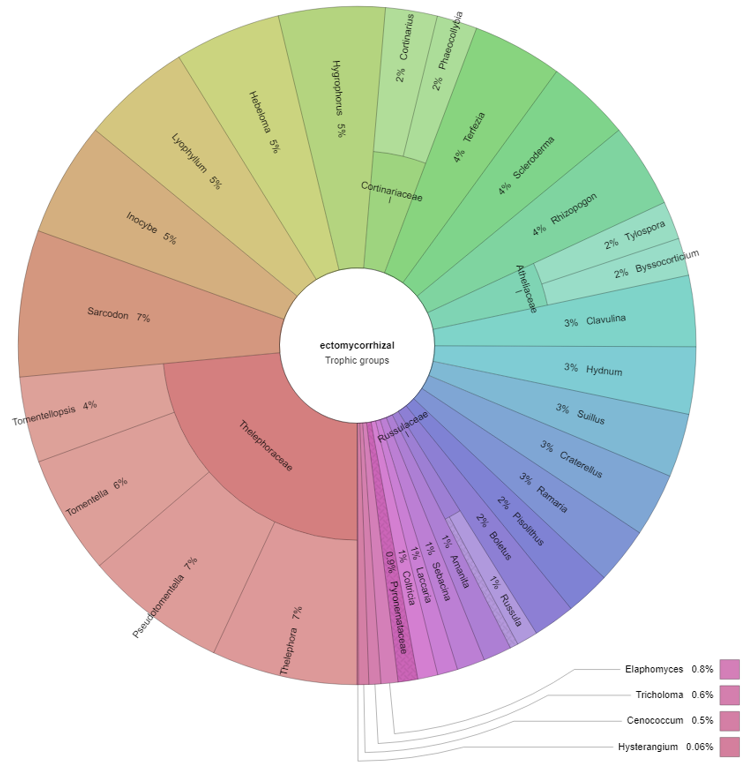


Sup fig. 1. Krona charts showing taxonomic classification of the ectomycorrhizal trophic groups found in all the treatments at genus level based on Põlme et al.^45^
